# Differences in the Expression of KIR, ILT Inhibitory Receptors, and VEGF Production in the Induced Decidual NK Cell Cultures of Fertile and RPL Women

**DOI:** 10.1155/2021/6673427

**Published:** 2021-05-04

**Authors:** Monika Kniotek, Aleksander Roszczyk, Michał Zych, Monika Szafarowska, Małgorzata Jerzak

**Affiliations:** ^1^Department of Clinical Immunology, Transplantation Institute, Medical University of Warsaw, Nowogrodzka 59, Warsaw, 02-006 Mazovian Voivodeship, Poland; ^2^Department of Gynecology and Gynecologic Oncology, Military Institute of Health Sciences, Szaserów 128, Warsaw, 04-141 Mazovian Voivodeship, Poland

## Abstract

**Results:**

KIR2DL1 and ILT-2 expression on idNK cells was higher in healthy women than in RPL patients. Sildenafil enhanced NKG2A expression in RPL patients. VEGF concentration was higher in fertile woman idNK cell cultures. idNK cells were more sensitive for necrosis in RPL than in fertile women. SC did not influence VEGF production or idNK cell apoptosis.

**Conclusions:**

A combination of hypoxia, IL-15, and AZA promotes the conversion of pbNK into idNK cells CD56^+^CD16^−^-expressing KIR receptors and produces VEGF. Alterations in KIR2DL1 and ILT-2 expression as well as impaired VEGF production were associated with RPL. SC affects NKG2A expression on RPL idNK cells. SC had no effect on VEGF release or idNK cell apoptosis.

## 1. Introduction

Decidual NK (dNK) cells are a distinct population of NK cells, homing the decidua and becoming the most abundant and important population of immune-competent cells in the human uterus. dNK cells markedly increase in number after ovulation and reach the peak during the luteal phase. If fertilization occurs, they continue to proliferate in the decidua during the first trimester of pregnancy [1–4]. In the first 3 months of pregnancy, NK cells represent 50-70% of decidual leukocytes [5] and play a crucial role in angiogenesis and spiral artery formation by secreting the following: VEGF (vascular endothelial growth factor), angiotensin-1 (Ang-1), angiotensin-2 (Ang-2), and placental growth factor (PLGF) [1]. VEGF-A (also known as VEGF) is the principal inducer of angiogenesis. Moreover, some of its important roles are to stimulate trophoblast proliferation, develop embryonic vasculature, and promote maternal and fetal blood cell growth during early stages of pregnancy [6]. Impaired VEGF production was found in the serum of RPL and preeclampsia patients [7, 8].

Almost 90% of decidual natural killer (dNK) cells phenotypically and functionally resemble the peripheral blood CD56^bright^ CD16^−^ NK cell subset, which comprises noncytotoxic, highly cytokine-producing cells. The lack of cytotoxicity is correlated with the expression of a unique repertoire of activating (KAR) and inhibitory (KIR) receptors. The remaining 10% of cells in this subset phenotypically and functionally resemble CD16^+^CD56^dim^ cells. The most important inhibitory receptors include CD94/NKG2A-B (CD159a-b), CD94/NKG2C-E (CD159c-e), KIR2DL1 (CD158a), and ILT-2 (CD85j) which recognize nonclassical human leukocyte antigens (HLA): HLA-C, HLA-E, HLA-F, and HLA-G, respectively, expressed on the trophoblast surface [2, 9]. The role of dNK cells is to interact with the invading extravillous trophoblasts and regulate trophoblast invasion. Strong evidence was presented to confirm that apoptosis and extracellular matrix degradation played an important role in this process, and leucocytes associated with this phenomenon were dNK cells and macrophages [10]. It was shown that the impaired expression of inhibitory and activating receptor repertoire was significantly different in women with recurrent pregnancy loss or pregnancy failure episodes [11–14]. Changes in KIR and KAR expression influenced the activation level of dNK cells and their function, including the secretion of angiogenic factors and cytokine production. It was demonstrated that a change in the activity of dNK cells, which led to pregnancy loss, was reflected by the higher activity of peripheral blood NK cells [15].

Sildenafil citrate is a PDE5 (phosphodiesterase type 5) competitive inhibitor that causes the accumulation of cGMP in cells [16]. Nitric oxide (NO) relaxes vascular smooth muscle through the cyclic guanosine monophosphate- (cGMP-) mediated pathway [17]. During normal pregnancy, the trophoblast releases nitric oxide (NO), which is a potent vasodilator. However, decreased NO release may occur in pregnancies complicated by preeclampsia or intrauterine growth restriction [18]. NO synthase isoforms were identified in the uterus [19]. Sildenafil citrate (Viagra) augmented the vasodilatory effects of NO by preventing the degradation of cGMP [20]. Sildenafil applied as vaginal suppositories improved uterine artery blood flow and sonographic endometrial thickness in patients with previous unsuccessful assisted reproductive cycles due to poor endometrial response [20–22]. Some authors pointed out that treatment with SC increased the production of VEGF and Ang-1 during cardiovascular ischemia and diabetic erectile dysfunction [6, 23]. According to accumulating evidence, sildenafil citrate could be applied in the treatment of various complications of pregnancy, including intrauterine growth restriction (IUGR) [18, 24–26], low birth weight [27], preeclampsia, or idiopathic recurrent pregnancy loss (RPL) [18, 20, 27–30]. Since the presence of PDE5 in lymphocytes was demonstrated by Tenor et al. [31], numerous studies have focused on the impact of sildenafil citrate on the immune system [32].

Moreover, it was reported that the elevation of cGMP led to the overexpression of constitutively active PKG, which might result in the phosphorylation and activation of the JNK pathway and promote the apoptosis of some immune and cancer cells [17]. Hayden et al. reported that nitric oxide induced apoptosis in rat and human pulmonary artery smooth muscle cells (PASMCs) through the Fas-FasL pathway, which was enhanced by cGMP accumulation [33]. Thus, SC might also influence the apoptosis of NK cells.

In our previous study, we demonstrated that the intravaginal application of sildenafil citrate for 3-6 days during the proliferative phase of the menstrual cycle significantly decreased the activity of pbNK cells and improved uterine artery blood flow. This phenomenon was correlated with successful pregnancy outcomes [20]. It is unknown whether the influence of SC on NK cell activity depends on the changes of KIR/KAR expression or the induction of apoptosis. Therefore, this study focused on determining the ability of sildenafil to change the repertoire of inhibitory receptors (KIR2DL1, NKG2A, ILT-2, and ILT-4), important in the development of immune tolerance to the embryo, expressed on induced decidual NK cells (idNK cells). The apoptosis of idNK cells, as well as their ability to release VEGF, was checked after SC treatment.

Due to considerable problems with the isolation of dNK cells from the endometrium, the model of induced decidual NK (idNK) cells was used in this study. Cerdeira et al. reported that pbNK cells cultured in a combination of hypoxia, TGF-*β*1, IL-15, and 5-aza-2′-deoxycytidine (AZA) might be transformed to the following phenotypes of idNK cells: CD16^−^CD56^+^ cells which present features similar to dNK cells, including VEGF-A production [9].

## 2. Material and Methods

### 2.1. Ethical Approval for the Use of Human Peripheral Blood

The women were informed of the aim of the study, and the best interests of the participating patients always outweighed those of the research. The study was approved by the Bioethics Committee of the Medical University of Warsaw (KB/192/2015). All measurements, interventions, and blood collections were performed after informed consent was obtained from each woman participating in the study under the bioethics committee-approved protocol. All data obtained from the subjects were confidential and accessible only to the investigative personnel.

### 2.2. Study Subjects

#### 2.2.1. Control Group

The control group consisted of 24 fertile women without a history of obstetric-gynecological and internal disorders. None of the subjects included in the control group reported any problems as regards conception. They all declared a normal course of pregnancy and delivery. Besides, none of the control subjects was treated for any internal disorders. Women using oral hormonal contraception and another hormonal treatment or women with hormonal intrauterine devices were excluded from the study. Transvaginal ultrasound scans were performed in all patients between days 3 and 5 of the menstrual cycle to confirm the normal morphology of the uterus, endometrium, and appendages. Fasting blood samples were collected from fertile women during the luteal phase (between days 16 and 25 of the menstrual cycle) in the morning.

#### 2.2.2. Study Group

The subjects enrolled in this study were volunteer participants. They were recruited from Mediva, Medical Center in Warsaw, between February 2016 and May 2017. One hundred and fifty patients with RPL were evaluated. However, 24 patients (aged 35 ± 4.4 years) with unexplained RPL were finally included in the study group. Recurrent pregnancy loss was defined according to the ASRM guideline as two or more consecutive spontaneous miscarriages before the 20th week of gestation [34]. Complete medical, surgical, and social histories were obtained in all cases. All the women with a history of RPL were investigated in terms of any identifiable causes of abortion. The patients included in the study presented no anatomic, genetic, microbiological, immunological, or hormonal causes of abortions. Transvaginal ultrasound, hysterosalpingography, or hysteroscopy did not reveal any abnormalities in the patients' uteri. Peripheral blood chromosome assessment confirmed normal karyotypes. All laboratory tests including hormonal assessment revealed no abnormalities. Besides, none of the subjects was treated for any internal disorders or had surgical interventions. The age and number of miscarriages are shown in Table 1. According to our study protocol, blood samples were collected from RPL patients, 6 months after the last miscarriage, so the immunological status of the patients had been normalized before the research. The blood was collected from the patients between days 16 and 25 of the menstrual cycle, as in our previous study [20].

### 2.3. Methods

#### 2.3.1. Peripheral Blood Mononuclear Cell and CD56^+^ Cell Isolation

Peripheral blood mononuclear cells (PBMC) were isolated from 20 ml of peripheral blood of 23 women with recurrent abortions and 24 healthy volunteers via Ficoll gradient centrifugation. After being washed twice in 0.9% Natrium Chloratum (Fresenius, Kabe), the cells were suspended in 1 ml of cold MACS buffer (0.5% BSA, 2 mM EDTA in PBS, Miltenyi Biotec, GmbH, Germany). The cells were counted and stained according to the manufacturer's instructions with the appropriate amount of CD56^+^ microbeads (20 *μ*l of CD56 microbeads per 10^7^ PBMC). After washing, the stained cells were separated with MidiMACS manual separator (Miltenyi Biotec, GmbH, Germany) according to the manufacturer's instructions (Miltenyi Biotec, GmbH, Germany). After isolation, we obtained approximately 2 × 10^6^ CD56-positive cells.

#### 2.3.2. Cell Culture

Isolated CD56-positive cells were cultured in 24-well plates (SPL Life Sciences Co., Ltd., Korea), in Opti-MEM Reduced Serum Media (Gibco, Life Technologist) containing 10% FCS (Sigma-Aldrich), 1 U/ml penicillin/streptomycin/100 *μ*g/ml (PAA), 2 mM glutamine (Sigma-Aldrich), 1 mM sodium pyruvate (Fluka), nonessential amino acids (Gibco, Thermo Fisher Scientific), 55 nM 2-mercapthoethanol, 10 ng/ml recombinant human IL-15 (Sigma-Aldrich), 2 ng/ml recombinant human TGF-*β*-1 (R&D), and 1 *μ*M 5-aza-2′-deoxycytidine (Sigma-Aldrich) in hypoxic (94% N_2_, 5% CO_2_, and 1% O_2_) environment [9]. The cells were cultured in 1 × 10^6^/ml concentration in two variants: with and without 400 ng/ml of sildenafil citrate (Sigma-Aldrich). The concentration of sildenafil used in the experiments equaled the blood concentration of sildenafil after the oral administration of 200 mg of Viagra in healthy men [35]. After 7 days of culturing, the cells were harvested for flow cytometry analysis.

#### 2.3.3. Flow Cytometry Staining

Mouse anti-human mAbs conjugated with appropriate fluorochromes (BD) used in this study are listed in Table 1. The BD™-CompBeads (Becton Dickinson) were stained with each of the fluorochrome-conjugated Abs separately and used as compensation controls.

NK cells collected from cultures were centrifuged at 1800 rpm for 10 min, and the supernatant was collected for VEGF determination. Subsequently, the cells were washed in Stain Buffer (1% FBS, Sigma-Aldrich, 0.09% sodium azide, Sigma-Aldrich, in PBS, Aqua Medica), suspended in 100 *μ*l of Stain Buffer and incubated with appropriate antibodies for 30 minutes on ice in the dark. After incubation, the cells were washed twice in Stain Buffer. KIR expression was analyzed on CD56^+^CD16^−^ cells. We performed staining controls, including fluorescence minus one (FMO) and isotype controls for KIR receptors to find the boundaries of cell populations (the gating strategy is presented in supplementary data: Figures S1 and S2).

Samples were acquired with BD FACS Canto II equipped with a 488 nm laser, a 633 nm laser, and a 405 nm laser. The data were analyzed with the FACSDiva 6.1.3. software.

*(1) Cell Culture Apoptosis Detection*. The collected cells in the amount of 1 × 10^5^ obtained by centrifugation of cell cultures were resuspended in 100 *μ*l of 0.1% NaN_3_ PBS (Sigma-Aldrich), and antibodies against surface markers for CD56 conjugated with Pe-Cy7 and CD16 with FITC were added (BD, USA). After 15 minutes of incubation in the dark at room temperature, the cells were washed twice in 1x Annexin V Binding Buffer (PBS, 1% Hepes, Sigma-Aldrich). Subsequently, the cells were labeled with 5 *μ*l of Bv421 Annexin V (BD, USA) to detect early apoptosis, and 5 *μ*l of 7AAD (BD, USA) to detect late apoptosis and necrotic cells. Then, the cells were incubated at room temperature for 5 minutes in the dark, and 400 *μ*l of Binding Buffer was added. The cells were acquired with FACS Canto II during the first hour after staining (detailed gating strategy is presented in supplementary data: Figure S3).

#### 2.3.4. VEGF Concentration Determination

The concentrations of VEGF in culture supernatants were measured with the double-antibody sandwich enzyme-linked immunosorbent assay (ELISA) according to the manufacturer's instructions to determine the level of cytokines. The concentrations of cytokines were calculated from the standard curve of linear regression according to the manufacturer's instruction (ELISA-kits, Sun Red, Biotechnology Company Co., Ltd., Shanghai, China). The levels of the sensitivity of ELISA-kits were VEGF, 9 pg/ml (<12% intra-assay range).

### 2.4. Statistical Analyses

All statistical analyses were performed with Graph Pad Prism 9.00, and the results were expressed as the mean and interquartile range. Normal distribution was determined with the Shapiro-Wilk test. Intergroup analyses were performed with the Wilcoxon matched paired test in the case of nonnormal distribution and the paired Student *t*-test for the normal distribution of samples. The unpaired *t*-test was used in the case of normal, and Mann–Whitney's *U*-test in the case of nonnormal distribution to determine the statistical significance in the control and study group. *p* values below 0.05 (*p* < 0.05) were considered statistically significant.

## 3. Results

### 3.1. Characteristics of the Studied Groups

The characteristics of the study group and multiparous controls, including age and the number of spontaneous pregnancy losses, are shown in Table 2. No difference was observed in the age between RPL patients and fertile women.

### 3.2. Comparison of the Expression of the Studied Receptors on idNK Cells in Fertile vs. RPL Women

Cultured in transformation media, CD56^+^CD16^+^ NK cells lost the expression of CD16 molecule similarly in both studied groups: CG idNK cells 75.67% ± 7.3% and RPL idNK cells 73.48% ± 11.6% (Figure S4 in supplementary data).

**Figure 1:**
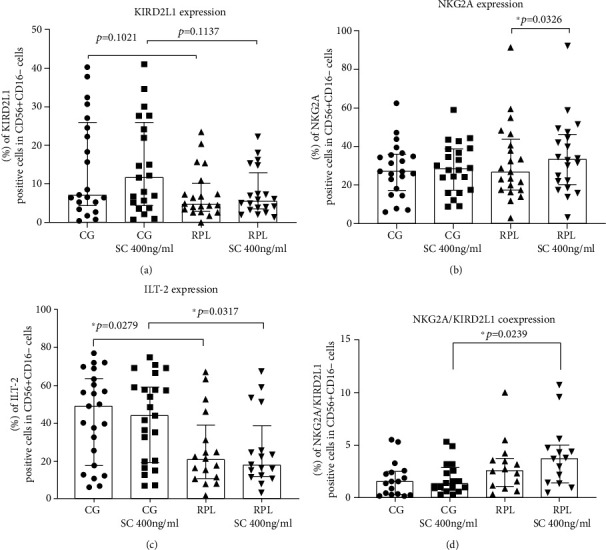
The expression of KIR receptors KIR2DL1, NKG2A, and ILT-2 and the coexpression of NKG2A with KIR2DL1 on idNK cells cultured with 400 ng/ml of sildenafil citrate (SC). CG: control group: (a) *n* = 22, (b) *n* = 24, (c) *n* = 23, and (d) *n* = 17; RPL: recurrent pregnancy loss patients: (a) *n* = 20, (b) *n* = 22, (c) *n* = 18, and (d) *n* = 14.

The expression of KIR2DL1 and ILT-2 on idNK cells was higher in fertile women than in RPL patients (Figure 1). We noticed that the appearance of ILT-2 after the conversion to idNK cells was characterized by very high diversity.

Notably, we observed a trend of the emphasized expression of double-positive cells CD56^+^CD16^−^KIR2DL1^+^NKG2A^+^ in RPL group compared to the healthy controls.

CD85d (ILT-4) countenance did not appear after pbNK cell conversion.

### 3.3. Influence of Sildenafil Citrate on the Conversion and Receptors of idNK Cells

SC improved NKG2A expression on idNK cells in RPL patients. Moreover, it sustained a higher expression of ILT-2 in the idNK cells of fertile women. Additionally, sildenafil improved the trend of the expression of scanty double-positive CD56^+^CD16^−^KIR2DL1^+^NKG2A^+^ cells in RPL group (Figure 1). SC did not exert a significant impact on the KIR2DL1 manifestation.

### 3.4. Cell Culture Apoptosis

**Figure 2:**
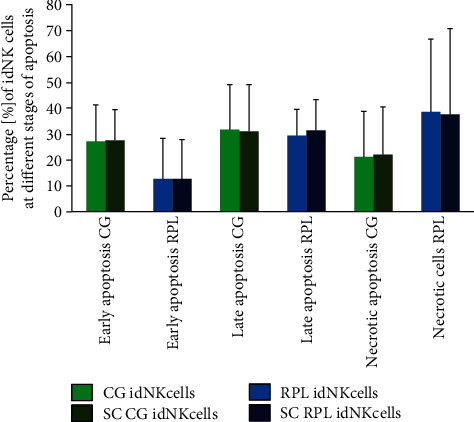
The stages of apoptosis of idNK cells after 7 days of culturing with and without 400 ng/ml of sildenafil citrate (SC). The results are presented as the mean + SD of the percentage of cells at different stages of apoptosis: green boxes, CG idNK cells in transformation media; deep green, CG idNK cells in transformation media supplemented with SC; blue boxes, RPL idNK cells in transformation media; and deep blue boxes, RPL idNK cells media supplemented with SC (CG: control group, *n* = 14; RPL: recurrent pregnancy loss women, *n* = 12).

The early apoptosis of idNK cells was measured as the expression of Annexin V. 7AAD and Annexin V staining was used to determine the late apoptosis, and 7AAD stained cells were considered necrotic cells. Sildenafil citrate was found not to affect the apoptosis of idNK cells at any stage (Figure 2).

### 3.5. VEGF Production by idNK Cells

**Figure 3:**
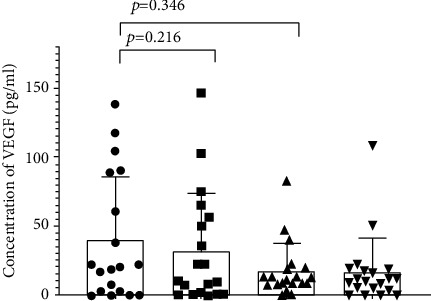
The level of VEGF in the culture supernatants of idNK cells (CG: control group, RPL: recurrent pregnancy loss women, and SC: sildenafil citrate; data shown as mean and SD).

VEGF level was rather emphasized in healthy fertile women in comparison with RPL patients' idNK cell cultures. Sildenafil weakens the production of VEGF in healthy women, but the results fall out of statistical significance (Figure 3).

## 4. Discussion

Recurrent pregnancy loss (RPL) is an important reproductive health problem, affecting 2-5% of couples. Despite extensive investigation, 40-50% of cases of RPL remain unidentified. Such cases are classified as idiopathic RPL [36]. Numerous authors suggested that the alterations of dNK cell subsets might be associated with recurrent pregnancy loss, implantation failures, and preeclampsia [12–14, 37]. Our previous research results showed that the treatment of RPL women with intravaginal sildenafil citrate resulted in diminished peripheral blood NK activity and positive pregnancy outcomes [19]. The objective of this study was to investigate if SC affected the expression of the inhibitory receptors of induced decidual NK cells. Due to a problem with collecting the endometrial tissue during spontaneous abortions, we decided to perform the study on pbNK converted to idNK cells according to the reports by Cerdeira et al. and Cavalli et al. [9, 38]. The researchers showed that the *ex vivo* manipulation of human peripheral blood NK (pbNK) cells by a combination of hypoxia, TGF*β*-1, and 5-aza-2′-deoxycytidine yielded cells with phenotypic and *in vitro* functional similarities to dNK cells, called idNK cells [9]. Cavalli et al. [38] reported that gene expression profiling revealed that CD56^+bright^ idNK cells derived *ex vivo* from human pbNK cells, and to a lesser extent, CD56^dim^ idNK cells were enriched in the gene expression signature, which distinguished dNK cells from pbNK cells [38]. Based on those data, we conducted a similar conversion with positive results. The population obtained after the conversion of pbNK cells contained ^+^KIR^+^CD56^bright^ CD16^−^ NK, similarly to the mentioned studies [9, 38].

In recent years, researchers demonstrated that the maternal KIR repertoire expressed on uterine NK cells might influence pregnancy outcomes [2–4]. We observed a tendency towards the lower expression of KIR2DL1 on idNK cells of RPL patients. The KIR2DL1 (CD158a) receptors are abundantly expressed on dNK cells, and the combination of maternal KIR and HLA-C molecules affects the depth of trophoblast invasion [3]. Faridi et al. [12] found that the percentage of KIR2DL1-positive dNK cells was diminished in RPL patients compared to multiparous dNK cells, which is in line with our findings. Similarly, Varla-Leftherioti et al. reported the limited gene expression of the inhibitory KIR repertoire in women with RPL compared to women with successful pregnancies [39]. Data presented by Hiby et al. confirmed that maternal inhibitory KIR2DL1 was associated with pregnancy disorders linked to inadequate placentation, and it negatively impacted fetal body weight [40]. Our study revealed no effect of SC on KIR2DL1 expression on the idNK cells of fertile or RPL women. No data are available on the influence of SC on the receptor, so we cannot compare our results.

NKG2A (CD159a) was another investigated receptor on idNK cells in our study due to the negative control of the cytotoxic potential of dNK cells [41]. The CD94/NKG2A heterodimer may operationally detect HLA-G1 and HLA-E molecules expressed on the trophoblast [42]. RPL patients and fertile women displayed a similar expression of NKG2A receptor on the idNK cells surface in our research, but SC improved the countenance of NKG2A in RPL group which may favor positive pregnancy outcomes, as NKG2A was shown to be involved in the regulation of uterine vascular adaptation to pregnancy, placental function, and transcriptome, as well as the regulation of fetal growth [43]. André et al. reported that blocking the inhibitory NKG2A receptor enhanced tumor immunity by promoting both natural killer (NK) and CD8+ T cell effector functions [44]. We observed the opposite effect of SC on the NKG2A receptor expression, which suggests the inhibitory action of SC on idNK cell cytotoxicity. The observed effect of SC may partially explain the impaired pbNK activity in RPL patients after SC treatment.

Ig-like transcript 2 (ILT-2, also known as CD85j or LILRB1) and ILT-4 (also known as CD85d or LILRB2) are the main HLA-G receptors on peripheral immune cells [45]. ILT-2 is expressed on T cells, B cells, monocytes, and dNK cells. It recognizes HLA-G antigens and highly conserved MHC class I proteins: beta-2 microglobulin and *α*3 chain of MHC I. The recognition of HLA-G by ILT-2 leads to the inhibition of dNK cell cytotoxicity [40, 46]. Our research demonstrated a reduction in the expression of ILT-2 on idNK cells in RPL patients in comparison with fertile women. The reduction of ILT-2 receptor may lead to the activation of NK cells as well as CD8 cells, and the secretion of IL-6, IL-8, and TNF-*α* due to impaired engagement with the HLA-G of the trophoblast [42, 47–49]. The lower expression of ILT-2 may explain widely observed enhanced pbNK cell activity in RPL patients. No data are available to confirm the influence of sildenafil on ILT-2 receptor abundance. In our study, sildenafil citrate sustained the difference in ILT-2 expression between the study and control group.

We could not detect the expression of ILT-4 on idNK cells, which is in line with the results of Fu et al. [50]. LeMaoult et al. claimed that HLA-G antigens upregulated the expression of ILT-2, ILT-3, and ILT-4 [51]. However, our idNK cultures did not contain HLA-G which might stimulate ILT-4 appearance.

Our results considering VEGF production by idNK cells are in line with the literature [7, 8]. However, contrary to the findings by Koneru et al. and Liu et al., sildenafil citrate rather decreased VEGF levels in fertile women and had no influence on idNK cell VEGF production in RPL patients [23, 52]. Lacchini et al. reported that the response to sildenafil may depend on VEGF or nitric oxide and cGMP pathway gene polymorphisms [53, 54].

Some authors pointed out that SC may have teratogenic and other possible toxic effects on mouse fetuses depending on the dose of the drug. SC at a dose of 40 mg/kg restricted the growth of the fetus [55]. Zhu et al. reported that PDE5 played a dominant role in regulating cGMP transitions that inhibited cell growth and controlled the susceptibility to apoptosis in the pulmonary endothelium [56]. Nevertheless, our specific flow cytometry analysis revealed that the drug did not affect idNK cell apoptosis, at neither early nor late stages, which confirmed the previous research results obtained by our group [57, 58].

To conclude, we found a lower percentage of KIR2DL1 and ILT-2 inhibitory receptors expressed on the idNK cells of patients with RPL compared to fertile women. Low KIR2DL1 was linked to insufficient placentation and fetal body weight. Decreased ILT-2 might impair HLA-G recognition and restrict fetal growth which could affect pregnancy outcomes.

We observed the influence of SC on the expression of NKG2A receptor inhibitory receptors on idNK cells, which may partially explain our previous findings suggesting that SC decreased NK cell activity in RPL patients. SC may act also on other immune mechanisms, e.g., on the production of cytokines, involved in the regulation of NK cell activity which was tested subsequently.

The present study is the first one that determines the receptors of idNK cells in RPL patients.

More research is needed to determine whether SC affects dNK or idNK cell function and their receptors.

## Figures and Tables

**Table 1:** Mouse anti-human antibodies with conjugated fluorochromes were used in the experiment. Phenotyping was performed in 4 color schemes in three tubes. Each tube included CD56 and CD16 to gate the population of interest: in tube 1, the induction of NK cells to idNK cells and killer immunoglobulin-like receptors were assessed, and in tube 2, ILT expression was checked.

Marker	Fluorochrome	Clone	Isotype control	Manufacturer	The volume of Abs per tube	Tubes
CD56	PE-Cy7	B159	—	Becton Dickinson	5 *μ*l	1, 2
CD16	FITC	3G8	—	Becton Dickinson	20 *μ*l	1, 2
CD158a/KIR2DL	PE	HP-3E4	Mouse BALB/c IgM	Becton Dickinson	20 *μ*l	1
CD159a/NKG2A	APC	Z199	Mouse IgG2b	Beckman Coulter	7 *μ*l	1
CD85j/ILT-2	PE	GHI/75	Mouse IgG2b	Becton Dickinson	20 *μ*l	2
CD85d/ILT-4	Alexa647	287219	Mouse IgG2a, *κ*	Becton Dickinson	5 *μ*l	2

**Table 2:** Characteristics of the studied groups.

	Fertile women (*n* = 24)	RPL patients (*n* = 24)
Age (years)	37.4 ± 1.9	35 ± 4.4
No. of clinical losses	0	3.7 ± 1.3

## Data Availability

The KIR.xlsx. and VEGF.xlsx data used to support the findings of this study are available from the corresponding author upon request.

## Abbreviations

Abs: Antibodies
APA: Antiphospholipid antibodies
APS: Antiphospholipid antibody syndrome
AZA: 5-Aza-2′-deoxycytidine
cGMP: Cyclic guanosine monophosphate
CG: Control group
dNK: Decidual NK cells
FMO: Fluorescence minus one
GMP: Guanosine monophosphate
idNK: Induced decidual NK cells
IL: Interleukin
ILT: Human inhibitory receptors Ig-like transcript
JNKs: C-Jun N-terminal kinases
KARs: Killer activation receptors
KIRs: Killer cell immunoglobulin-like receptors
NO: Nitric oxide
NOS: Nitric oxide synthase
PBS: Phosphate-buffered saline
PCOS: Polycystic ovary syndrome
PDE-Is: Phosphodiesterase inhibitors
PDEs: Phosphodiesterases
PGE: 2–Prostaglandin-2
PKG: Protein kinase G
PRL: Prolactin
RPL: Recurrent pregnancy loss
RSA: Recurrent spontaneous abortion
SC: Sildenafil citrate
uNK: Uterine NK cells
SG: Study group
TSH: Thyroid-stimulating hormone
VEGF: Vascular endothelial growth factor.

## Supplementary Materials

Supplementary Materials Figure S1: analysis of NK cells before culturing in transformation media. Gating strategy of NK cells. (a) Left dot plot: lymphocytes were identified on FSC/SSC, cells 50 × 10^3^ to 150 × 10^3^ on FSC and below 200 × 10^3^ on SSC; right dot plot: CD56^+^ CD16^−^ NK cells were identified as positive events with Pe-Cy7 fluorescence above 10^3^ and lower than 10^3^ with FITC fluorescence. CD56^+^ CD16^+^ cells were identified as positive events with FITC fluorescence above 10^3^ on a logarithmic scale. (b) Gated CD56^+^ CD16^−^ and CD16^+^CD56^−^ cells were analyzed for CD159a expression, and APC-positive cells were identified with APC fluorescence above 2 × 10^2^ on a logarithmic scale, and for CD158a expression, PE-positive cells were identified with PE fluorescence above 2 × 10^2^ on a logarithmic scale. Figure S2: gating strategy of idNK cells. (a) NK cells after culturing in transformation media; NK lymphocytes were identified on FSC/SSC dot plot as cells 50 × 10^3^ to 50 × 10^3^ on FSC and below 200 × 10^3^ on SSC. (b) CD56^+^ CD16^−^ idNK cells were identified as positive events with Pe-Cy7 fluorescence above 10^3^ and lower than 10^3^ with FITC fluorescence on a logarithmic scale. (c) Gated CD56^+^ cells were analyzed for the presence of nonspecific staining with isotype controls for CD158a-PE and CD159a-APC markers. (d) Gated CD56^+^ cells were analyzed for CD158a^+^ CD159a^+^ expression; positive events were gated as cells with fluorescence above 10^3^ for PE and above 10^3^ for APC on a logarithmic scale. (e) Staining with isotype controls for CD85j and CD85d molecules. (f) CD85j- or CD85-positive idNK cells were identified as idNK events with PE or APC fluorescence above 10^3^ on a logarithmic scale. Figure S3: determination of the apoptosis of induced decidual NK cells (isolated NK cells cultured for 7 days in transformation media). (a) Doublet exclusion. (b) Lymphocyte gating: lymphocytes were gated as cells on FSC/SSC dot plot as cells within 50 × 10^3^ to 150 × 10^3^ on FSC and below 200 × 10^3^ on SSC. (c) CD56^+^ CD16^−^ idNK cells were gated as cells with PE-Cy7 fluorescence above 10^3^ (on the *y*-axis). (d) The analysis of the apoptosis of idNK cells was performed with Annexin V Bv421 and 7AAd staining. Necrotic cells were identified as cells with strong red and violet fluorescence (above 10^5^ decades on PerCP-Cy5.5 fluorescence); late apoptosis as events positive for red and violet fluorescence (above 10^5^ decades on PerCP-Cy5.5 fluorescence and 10^3^ decades on Pacific Blue); idNK in early apoptosis was identified as positive events on violet fluorescence above 10^3^ decades on Pacific Blue axis; live cells were identified as events below 10^5^ decades on PerCP-Cy5.5 fluorescence and below 10^3^ on Pacific Blue. Figure S4: the percentage of CD56^+^ CD16^−^ cells among NK cells before culturing in transformation media (NK CG, and NK RPL) and after culturing (idNK CG, idRPL). Data are shown as the median ± SD.
