# Supplementary material for: Differences in the Expression of KIR, ILT Inhibitory Receptors, and VEGF Production in the Induced Decidual NK Cell Cultures of Fertile and RPL Women
Source: Biomed Res Int. 2021 May 4;2021:6673427. doi: 10.1155/2021/6673427 (PMC8112925; doi:10.1155/2021/6673427)
Supplement: Supplementary Materials — Figure S1: analysis of NK cells before culturing in transformation media. Gating strategy of NK cells. (a) Left dot plot: lymphocytes were identified on FSC/SSC, cells 50 × 103 to 150 × 103 on FSC and below 200 × 103 on SSC; right dot plot: CD56+ CD16− NK cells were identified as positive events with Pe-Cy7 fluorescence above 103 and lower than 103 with FITC fluorescence. CD56+ CD16+ cells were identified as positive events with FITC fluorescence above 103 on a logarithmic scale. (b) Gated CD56+ CD16− and CD16+CD56− cells were analyzed for CD159a expression, and APC-positive cells were identified with APC fluorescence above 2 × 10^2^ on a logarithmic scale, and for CD158a expression, PE-positive cells were identified with PE fluorescence above 2 × 10^2^ on a logarithmic scale. Figure S2: gating strategy of idNK cells. (a) NK cells after culturing in transformation media; NK lymphocytes were identified on FSC/SSC dot plot as cells 50 × 103 to 50 × 103 on FSC and below 200 × 103 on SSC. (b) CD56+ CD16− idNK cells were identified as positive events with Pe-Cy7 fluorescence above 103 and lower than 103 with FITC fluorescence on a logarithmic scale. (c) Gated CD56+ cells were analyzed for the presence of nonspecific staining with isotype controls for CD158a-PE and CD159a-APC markers. (d) Gated CD56+ cells were analyzed for CD158a+ CD159a+ expression; positive events were gated as cells with fluorescence above 103 for PE and above 103 for APC on a logarithmic scale. (e) Staining with isotype controls for CD85j and CD85d molecules. (f) CD85j- or CD85-positive idNK cells were identified as idNK events with PE or APC fluorescence above 103 on a logarithmic scale. Figure S3: determination of the apoptosis of induced decidual NK cells (isolated NK cells cultured for 7 days in transformation media). (a) Doublet exclusion. (b) Lymphocyte gating: lymphocytes were gated as cells on FSC/SSC dot plot as cells within 50 × 103 to 150 × 103 on FSC and below 200 × 103 on SSC. (c) CD56+ CD16 [file 6673427.f1.docx]

**Differences in the expression of KIR, ILT inhibitory receptors, and VEGF production in the induced decidual NK cell cultures of fertile and RPL women**

Monika Kniotek^1^, Aleksander Roszczyk^1^ , Michał Zych^1^, Monika Szafarowska^2^, Małgorzata Jerzak^2^

^1^Department of Clinical Immunology, Transplantation Institute, Medical University of Warsaw, Nowogrodzka 59, Warsaw, 02-006, Mazovian Voivodeship, Poland

^2^Department of Gynecology and Gynecologic Oncology, Military Institute of Health Sciences, Szaserów 128, Warsaw, 04-141, Mazovian Voivodeship, Poland

Corresponding author: Monika Kniotek

Department of Clinical Immunology,

Transplantation Institute,

Medical University of Warsaw

Nowogrodzka 59, Warsaw, 02-006,

Mazovian Voivodeship, Poland

e-mail address: [monika.kniotek@wum.edu.pl](mailto:monika.kniotek@wum.edu.pl),

phone number: +48 22 502 12 62, +48 666-066-930

ORCID no: 0000-0002-1510-7236

a)


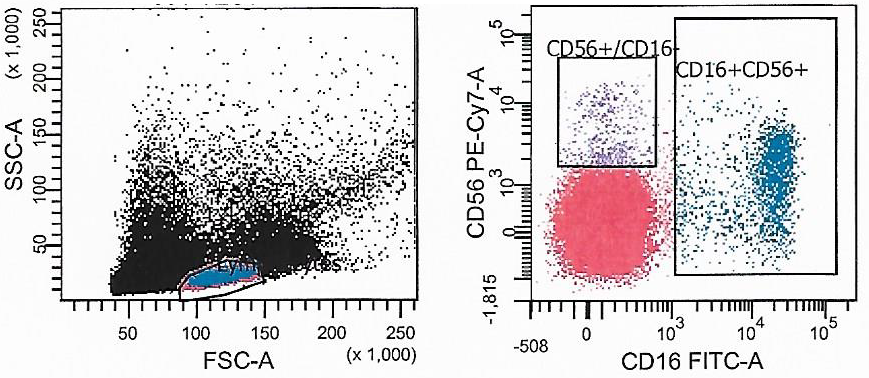


b)


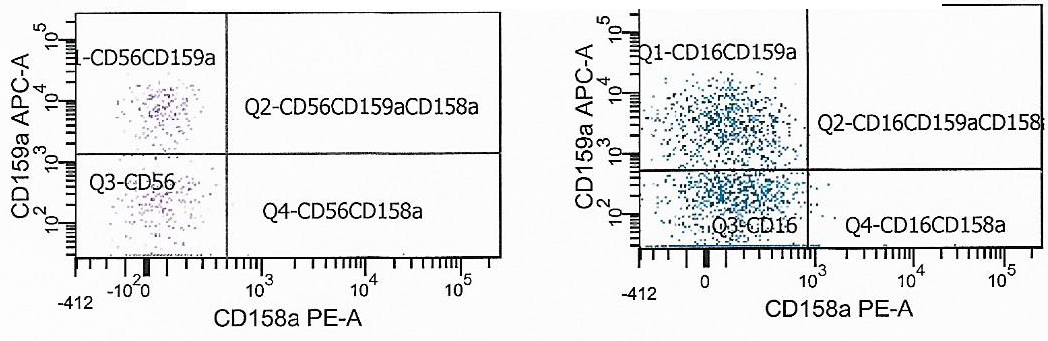


Fig. S1. Analysis of NK cells before culturing in transformation media. Gating strategy of NK cells, a) left dot plot: lymphocytes were identified on FSC/SSC, cells 50-150 x 10^3^ on FSC and below 200 x 10^3^ on SSC; right dot plot: CD56^+^ CD16^-^  NK cells were identified as positive events with Pe-Cy7 fluorescence above 10^3^ and lower than 10^3^ with FITC fluorescence, CD56^+^ CD16^+^ cells were identified as positive events with FITC fluorescence above 10^3^ on a logarithmic scale, b) gated CD56^+^ CD16^-^ and CD16^+^CD56^-^ cells were analyzed for CD159a expression, APC-positive cells were identified with APC fluorescence above 2x10^2^ on a logarithmic scale, and for CD158a expression, PE-positive cells were identified with PE fluorescence above 2x10^2^ on a logarithmic scale.

1. b)


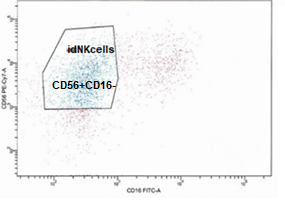

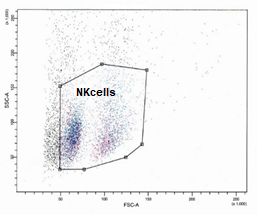


c) d)


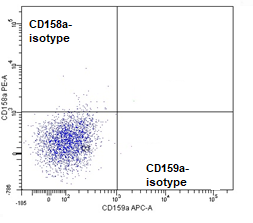

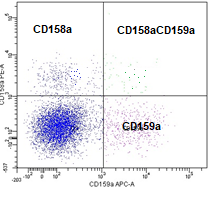


e) f)


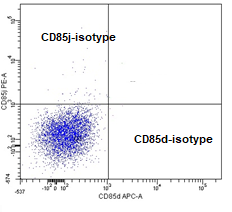


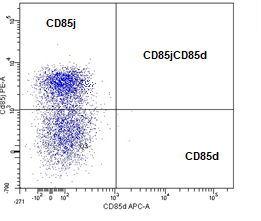


Fig. S2. Gating strategy of idNK cells, a) NK cells after culturing in transformation media; NK lymphocytes were identified on FSC/SSC dot plot as cells 50-150 x 10^3^ on FSC and below 200 x 10^3^ on SSC, b) CD56^+^ CD16^-^ idNK cells were identified as positive events with Pe-Cy7 fluorescence above 10^3^ and lower than 10^3^ with FITC fluorescence on a logarithmic scale, c) gated CD56^+^ cells were analyzed for the presence of nonspecific staining with isotype controls for CD158a-PE and CD159a-APC markers, d) gated CD56^+^ cells were analyzed for CD158a^+^ CD159a^+^ expression, positive events were gated as cells with fluorescence above 10^3^ for PE and above 10^3^ for APC on a logarithmic scale, e) staining with isotype controls for CD85j and CD85d molecules, f) CD85j or CD85 positive idNK cells were identified as idNK events with PE or APC fluorescence above 10^3^ on a logarithmic scale.

1.
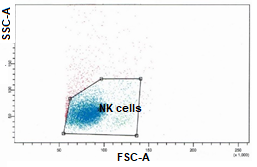
 b)


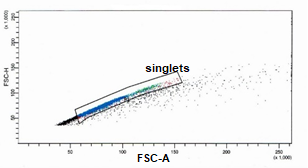


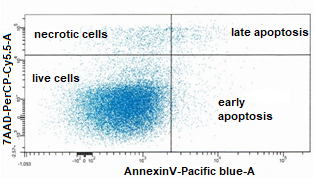
 c) d)


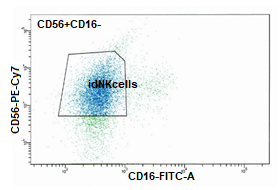


Fig. S3. Determination of the apoptosis of induced decidual NK cells (isolated NK cells cultured for 7 days in transformation media), a) doublet exclusion, b) lymphocyte gating – lymphocytes were gated as cells on FSC/SSC dot plot as cells within 50-150 x 10^3^ on FSC and below 200 x 10^3^ on SSC, c) CD56^+^ CD16 - idNK cells were gated as cells with PE-Cy7 fluorescence above 10^3^ (on the y-axis), d) the analysis of the apoptosis of idNK cells was performed with annexin-V-Bv421 and 7AAd staining. Necrotic cells were identified as cells with strong red and violet fluorescence (above 10^5^ decades on PerCP-Cy5.5 fluorescence); late apoptosis as events positive for red and violet fluorescence (above 10^5^ decades on PerCP-Cy5.5 fluorescence and 10^3^ decades on Pacific Blue); idNK in early apoptosis was identified as positive events on violet fluorescence above 10^3^ decades on Pacific Blue axis; live cells were identified as events below 10^5^ decades on PerCP-Cy5.5 fluorescence and below 10^3^ on Pacific Blue.

Fig. S4. The percentage of CD56^+^CD16^-^ cells among NK cells before culturing in transformation media (NK CG, NK RPL) and after culturing (idNK CG, idRPL). Data are shown as the median ± SD.
